# A Comparative dose and image quality assessment of a portable, multi‐modality extremity imaging system for austere environments

**DOI:** 10.1002/mp.70574

**Published:** 2026-07-25

**Authors:** Xiangyu Yang, Edward J. Stafford, Hadley A. DeBrosse, Yi Wei, Adam Smith, David E. Hintenlang, Nathaniel Bates

**Affiliations:** ^1^ Department of Radiology The Ohio State University Wexner Medical Center Columbus Ohio USA; ^2^ Department of Orthopaedics The Ohio State University Wexner Medical Center Columbus Ohio USA

**Keywords:** DDR, field imaging, portable radiography

## Abstract

**Background:**

Rapid technological innovation in diagnostic imaging requires timely, evidence‐based safety and quality assessment to ensure optimal patient care. This study evaluates a novel multi‐modality extremity imaging system (OXOS MC2) designed for deployment in remote and austere environments.

**Purpose:**

To systematically evaluate the radiation safety and image quality characteristics of the MC2 system in its three imaging modalities (radiography, fluoroscopy, and Dynamic Digital Radiography [DDR]), and to compare them with two conventional, single‐modality reference systems: a portable radiographic unit and a mobile C‐arm fluoroscopic unit.

**Method:**

Radiation dose and image quality were assessed for four extremity regions (hand, shoulder, ankle, knee). Patient dose metrics (Entrance Air Kerma [EAK] and Entrance Air Kerma Rate [EAKR]) were estimated using clinical techniques and measured primary radiation output. Operator dose (effective dose equivalent, H_E_) was estimated using a “virtual badge” method based on measured scatter map, leakage rate, and clinical setups provided by practicing orthopedic surgeons. Spatial resolution was quantified by the Modulation Transfer Function (MTF) using the slant‐edge method. Contrast performance was analyzed using a Gammex contrast‐detail phantom. The image receptor input dose (IRID) and input dose rate (IDRIR) were also measured. Dose comparisons were summarized descriptively due to the small sample size. Image quality comparisons were performed using generalized linear models with Bonferroni correction for multiple comparisons (statistical significance set at *p* < 0.0019 for each individual test).

**Results:**

The MC2 system demonstrated substantially lower patient EAK compared to the radiographic reference system (14%–59% of the reference). Although EAKR varied relative to the reference fluoroscopic system (15%–245% of the reference) due to differences in setup, the MC2's maximum AKR (3.53 mGy/min) remained well below the regulatory limit (44 mGy/min), indicating a negligible risk of radiation‐induced skin injury for the patient. The IRID and IDRIR exhibited trends similar to the EAK and EAKR. Operator doses for the MC2 were consistently lower than those of both reference systems, with the H_E_ at 9%–45% of the radiographic reference and 2%–80% of the fluoroscopic reference. Patient and operator doses of DDR were comparable to those of fluoroscopy. The MC2 system showed consistent image quality across all three imaging modalities. Its spatial resolution (1.8–1.9 lp/mm) was comparable to the fluoroscopic reference system (1.8–1.9 lp/mm, *p* = 0.92) but significantly lower than the radiographic reference system (2.1–2.2 lp/mm, *p* < 0.001). Despite delivering a lower dose to the detector, the MC2 demonstrated no significant difference in low‐contrast performance (all p ≥0.11). Handheld operation showed no significant impact on image quality.

**Conclusion:**

The OXOS MC2 is a safe and versatile imaging system capable of meeting various extremity imaging needs in remote and austere environments. With substantially reduced radiation dose and improved portability, it provides a robust alternative to conventional portable radiographic systems. While the MC2 system exhibits compromises in spatial resolution and beam hardness, its unique multi‐modality capabilities enable the field deployment of previously inaccessible fluoroscopic and DDR imaging, thereby greatly enhancing patient access to advanced imaging techniques in resource‐limited settings.

## 1 INTRODUCTION

Advances in X‐ray technology have enabled compact, versatile imaging systems that integrate multiple modalities, improve low‐dose and digital processing, and expand clinical applications beyond traditional niches.^1^, ^2^ Over the past few years, several innovative X‐ray systems that do not strictly adhere to conventional modality classifications have obtained regulatory approval and entered European and North American markets, with additional systems anticipated in the near future.^3^, ^4^, ^5^, ^6^, ^7^ While these novel systems offer possibilities for new clinical applications, they also present pressing challenges for the radiologic community to address new questions regarding their safe use, clinical indication, standardization, and regulatory control.

This study provides a systematic evaluation of a new multi‐modality portable X‐ray imaging system in comparison with conventional imaging platforms. The assessment was specifically set in the context of an underserved clinical scenario: delivering state‐of‐the‐art imaging techniques to remote and austere environments, including battlefield medicine, disaster response, ship‐based care, remote community clinics, and temporary relief centers. These environments often lack stable power grids and suffer from severe space constraints. The absence of hospital‐based infrastructure frequently makes portable imaging the only viable option.

Orthopedic injuries represent the predominant medical demand encountered in these austere settings. For instance, non‐combat fractures pose a major threat to military readiness, consistently surpassing combat injuries in incidence and resource burden.^8^, ^9^, ^10^ Musculoskeletal injuries were also identified as the leading cause of morbidity following major natural disasters, such as earthquakes.^11^ Onsite diagnostic and treatment options are usually limited. Imaging serves the primary purposes of rapid diagnosis and triage, as well as guiding stabilization procedures prior to transfer for definitive care. Therefore, multi‐modality portable imaging systems, such as the one evaluated in this study, offer distinct advantages over conventional single‐modality systems by broadening the range of diagnostic and treatment options available to medical personnel.

In addition to the conventional radiographic and fluoroscopic modalities, this new system also features Dynamic Digital Radiography (DDR, also known as serial radiography), a relatively recent imaging modality introduced in the early 2000s. A typical DDR implementation exposes a wireless digital detector to a series of rapid X‐ray pulses, from which subsequent image reconstruction produces a dynamic sequence of anatomical structures in motion. While the exposure pattern is analogous to that of pulsed fluoroscopy, the maximum duration of a DDR series is limited (typically ≤ 20 s).^6^ Consequently, it is better suited for short diagnostic tasks rather than prolonged interventional guidance.

With frame rates comparable to conventional pulsed fluoroscopic systems (2 to 15 pulses per second, PPS), DDR provides sufficient temporal resolution for the dynamic examination of physiological motion. Furthermore, this modality can be applied in various patient orientations, including weight‐bearing positions. It therefore holds great potential to become a valuable orthopedic tool that can bridge the gap between conventional static radiography, which lacks dynamic information, and fluoroscopy, which is usually performed with the patient in a recumbent position. However, current DDR literature has primarily focused on body imaging systems optimized for dynamic chest imaging,^6^ with little research exploring other potential applications. This work addresses this deficiency by providing essential radiation dose and image quality data for an orthopedic implementation specifically designed for extremity imaging. These data are critical for clinical decision‐making and facilitating safety assessments in this emerging field. Additionally, these findings offer a useful reference for future safety and compliance evaluations, thereby providing a foundation for broader discussions on radiation protection and regulatory oversight of innovative imaging equipment.

## 2 MATERIAL AND METHODS

The imaging system characterized in this study is a portable extremity X‐ray device that recently received United States Food and Drug Administration (FDA) 510(k) clearance (MC2, OXOS Medical, Atlanta, GA, USA). This device integrates three imaging modalities (radiography, fluoroscopy, and DDR) into a compact unit weighing 10.5 kg sized to fit within a small suitcase. Its battery‐powered operation allows for consistent functionality in environments with unstable power. Collectively, these features make the system particularly well‐suited to meet the versatile clinical demands of field deployment in austere environments.

**FIGURE 1:**
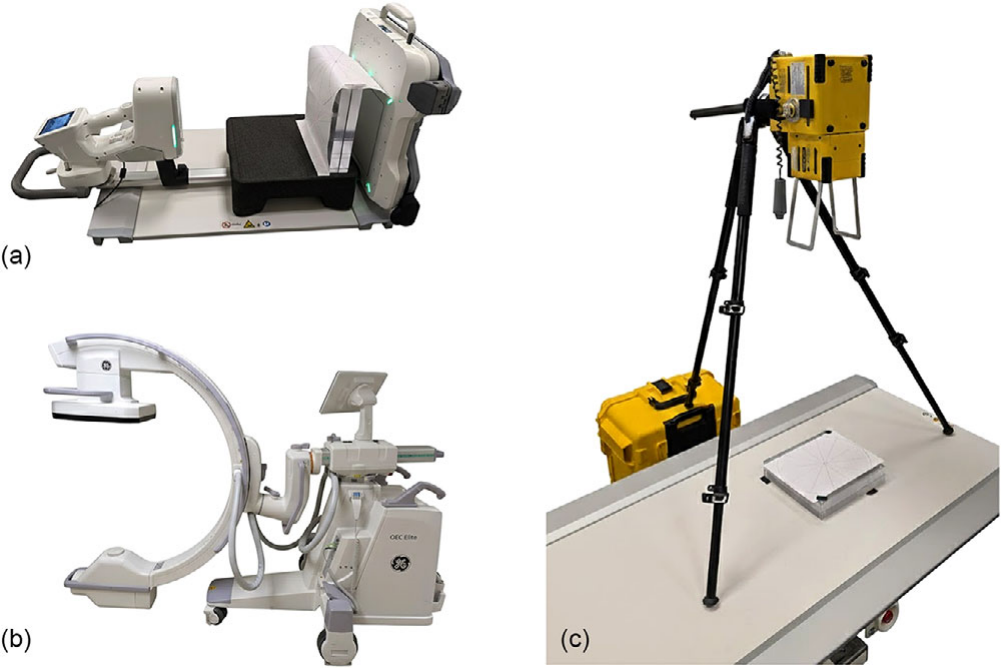
(a) The OXOS MC2 portable multi‐modality extremity X‐ray system, mounted on the lower‐extremity stand in the horizontal configuration. (b) The fluoroscopic reference system (OEC Ergo‐C). Only the C‐arm unit was photographed. The mobile workstation was not included. (c) The radiographic reference system (MinXray TR90BH), mounted on a tripod.

The MC2 system consists of a lightweight X‐ray emitter (nominal focal spot 0.8, total filtration 3.5 mm Al) and a wireless, flat panel amorphous silicon (a‐Si) Thin Film Transistor (TFT) detector coupled with cesium iodide (CsI) scintillator crystals (Mercu 0909X, iRay Group, Shanghai, China). The detector features a pixel pitch of 139 µm, real‐time imaging up to 30 frames per second (fps), and built‐in infrared beacons for source‐to‐image distance (SID) tracking and positioning (Figure 1a). The X‐ray emitter can be activated either while handheld (disabled for fluoroscopic imaging) or when mounted on a general‐purpose clinical cart (SID: 45 cm) or a lower‐extremity stand (SID: 55 cm). Interlock systems disable X‐ray production when the SID exceeds 80 cm or the source‐to‐skin distance (SSD) falls below 30 cm. Both DDR and fluoroscopy utilize pulsed X‐ray exposures at rates of 2.5 or 5 PPS. The maximum duration of a single image series is limited to 20 s, with a minimum interval of 40 s between consecutive 20‐second series (corresponding to a maximum duty cycle of 33.3%).

The two conventional systems used as references for comparison were a portable radiographic system (TR90BH, MinXray, Northbrook, IL, USA, nominal focal spot 0.8, total filtration: minimum 3.0 mm Al; Figure 1c) and a mobile C‐arm fluoroscopic system (OEC Ergo‐C with 31 cm flat panel detector, GE Healthcare, Chicago, IL, USA, nominal focal spot 0.3, total filtration 6.8 mm Al; Figure 1b). The portable radiographic system was selected because it represents the current benchmark for imaging in remote locations, given its widespread use by emergency responders and military medical services. Conversely, the C‐arm fluoroscopic system was included because its routine utilization in hospital‐based orthopedic procedures, coupled with its general unavailability in the field, underscores a substantial gap in the field care of orthopedic injuries.

The reference radiographic system is not equipped with a standard image detector. To minimize bias arising from detector characteristics, a wireless a‐Si/CsI radiographic detector from the same manufacturer was employed in this study (Venu 1717x, iRay Group, Shanghai, China). This detector features an identical pixel pitch (139 µm) and a larger format but does not support real‐time imaging. The reference fluoroscopic system utilizes a built‐in real‐time Complementary Metal‐Oxide‐Semiconductor (CMOS) detector with a pixel pitch of 198 µm and a frame rate of 30 fps. All image analyses were conducted on raw images derived directly from the detector without further post‐processing.

### 2.1 Primary, scatter, and leakage radiation measurement

Primary, scatter, and leakage radiation were systematically measured using a calibrated solid‐state dosimeter (X2, RaySafe, Hovås, Sweden). Primary radiation output and beam quality (half‐value layer, HVL) were measured across a range of tube voltages from 40 to 80 kilovolts (kV). The output data were fitted to a power‐law function of tube voltage (kVp). The resulting model was used for interpolation in patient dose estimation.

Scatter radiation was generated using an American National Standard Institute (ANSI) extremity phantom placed at the patient position simulating clinical setups.^12^ Three‐dimensional scatter maps were acquired at 35, 65, and 95 cm from the center of the phantom, at various polar and azimuthal angles in 30° increments. Cubic interpolation was employed on the angular distribution of scatter radiation in operator dose estimation. Because the MC2 system's clinical cart and lower‐extremity stand have different SIDs, scatter distributions were mapped in both an upright configuration (mounted on the clinical cart) and a horizontal configuration (mounted on the lower‐extremity stand laid on a patient table).

Tube leakage was measured around the X‐ray tubes at the maximum voltage relevant to extremity imaging (80 kVp) with the primary beam blocked by a lead sheet. Measurements were taken only at locations readily accessible to the survey meter probe. The highest leakage rate, regardless of polar or azimuthal angle, was used for operator dose estimation. While this approach likely overestimates the leakage contribution to the operator dose, its impact on the overall conclusions is minimal, as demonstrated by the data presented in the following section, due to the inherently low leakage levels.

### 2.2 Patient and operator dose estimate

**FIGURE 2:**
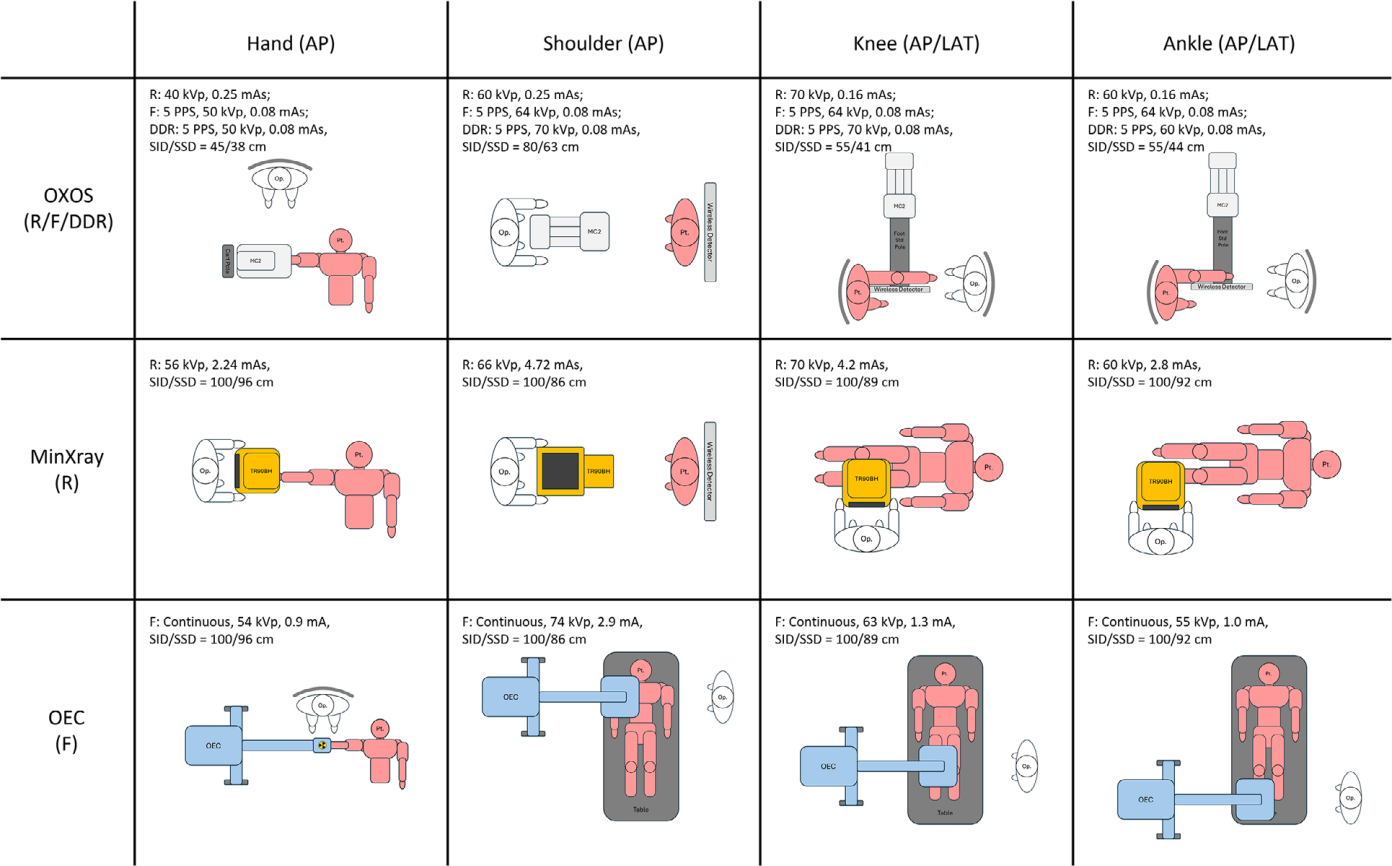
Illustration of patient set‐ups and exposure techniques of the system under study (OXOS) and two reference systems (MinXray and OEC). Handheld fluoroscopy is not currently approved for clinical use in the United States. Therefore, the estimated shoulder fluoroscopic dose for the system under study (OXOS) represents a hypothetical scenario evaluated solely for academic and investigational purposes. AP: the anteroposterior view; DDR: Dynamic Digital Radiography; F: the fluoroscopic operation mode; LAT: the lateral view; R: the radiographic operation mode; SID: source‐to‐image distance; SSD: source‐to‐skin distance.

While the mapping of primary and secondary radiation provides essential characterization of imaging equipment from a medical physics perspective, practical risk assessment and safety decisions made by clinicians are often guided by patient and staff dose metrics such as Entrance Air Kerma Rate (EAKR) and the effective dose equivalent (H_E_). Accordingly, several patient and operator dose metrics were estimated for four anatomical regions pertinent to extremity imaging: the hand, shoulder, ankle, and knee. The corresponding patient setups and technical parameters were summarized in Figure 2.

Patient setups were selected based on feedback from four practicing orthopedic surgeons with >30 combined years of clinical experience. Given that the MC2 system is not yet widely available, the setups for this system represent generalized procedures of the corresponding anatomical regions rather than established best practice protocols for specific clinical tasks. Exposure parameters for the MC2 system and the radiographic reference system were obtained from the manufacturer's recommendations in the corresponding user manuals. For the fluoroscopic reference system, which uses Automatic Exposure Rate Control (AERC) to determine exposure parameters in clinical practice, average clinical exposure techniques were obtained from relevant clinical cases archived in our institutional Picture Archiving and Communication System (PACS).

Entrance Air Kerma (EAK) and EAKR were chosen as metrics of patient dose for radiographic and fluoroscopic procedures, respectively. The Dose‐Area Product (DAP) was also calculated for comparison with published literature and regulatory limits. DDR was treated in a manner analogous to fluoroscopy due to similarities in temporal exposure patterns.

**FIGURE 3:**
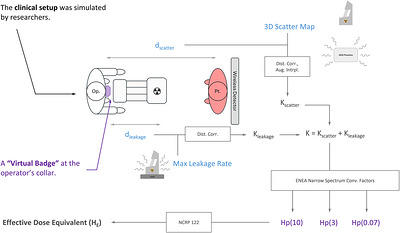
Illustration of the “virtual badge” method for occupational dose estimation. Quantities in blue text were obtained through experimental measurements. Quantities in purple text represent personal dose monitoring metrics commonly reported by radiation badges. K_scatter_ and K_leakage_ are the scatter and leakage air kerma, respectively. K is the total air kerma.

Occupational dose to the operator was estimated using a “virtual badge” method (Figure 3), in which the air kerma at the location corresponding to a collar badge worn by an operator in a simulated clinical setup was calculated based on measured scatter radiation and tube leakage. This value was then converted to the deep, shallow, and lens dose equivalents (Hp(10), Hp(0.07), and Hp(3), respectively) using narrow‐spectrum conversion factors proposed by the Italian National Agency for New Technologies (ENEA).^13^ Estimates of H_E_, the metric currently used in the United States radiation protection practices, were calculated for an operator wearing a protective apron by dividing Hp(10) by the appropriate conversion factor provided in Section 4.3 of the National Council on Radiation Protection and Measurements (NCRP) Report No. 122.^14^ Hp (0.07) and Hp(3) were retained as estimates of the doses to the skin and the lens of the eye, respectively.

While not directly quantifying personnel doses, the image receptor input dose (IRID) and input dose rate (IDRIR), as defined in the AAPM Task Group Report 272, are closely related to the signal‐to‐noise ratio (SNR) and, consequently, the low‐contrast performance of many imaging systems.^15^ These metrics were measured for their respective clinical configurations, with the detailed measurement methodology described in the Supporting Information.

### 2.3 Image quality assessment

High‐contrast spatial resolution was assessed by measuring the Modulation Transfer Function (MTF) along both in‐plane directions using safety razor blades placed on the surface of the image detector housing, with no other materials in the beam path. Images of the blade edges were analyzed using the slant‐edge method.^16^ The spatial frequency corresponding to 10% of the smoothed MTF curve was reported as the system's limiting spatial resolution.

**FIGURE 4:**
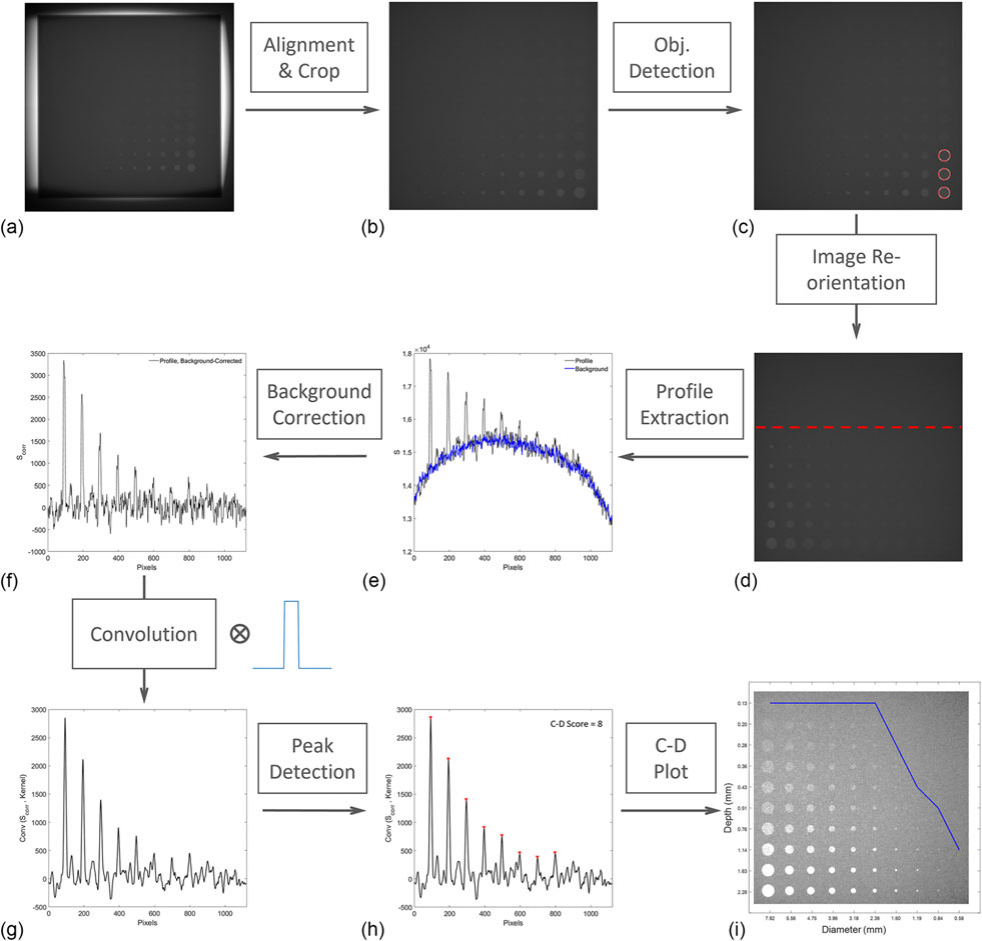
Illustration of the semi‐automated contrast‐detail analysis algorithm.

Low‐contrast detectability was evaluated using a contrast‐detail phantom consisting of a 10 × 10 matrix of holes with varying diameters and depths drilled into an aluminum block (Model 1511, Gammex, Middleton, WI, USA).^17^ The phantom was placed on the surface of the image detector housing, with no other materials in the beam path. A contrast‐detail curve was generated by plotting the depth of detectable holes as a function of their diameter. To minimize reader bias, a custom‐built, semi‐automated MATLAB script (R2024b, Mathworks, Natick, MA, USA) was developed for image analysis. (Figure 4).

### 2.4 Statistical analysis

Given the limited number of extremity protocols studied, neither parametric nor non‐parametric tests would have yielded sufficient statistical power to detect a true difference in operator or patient dose. Therefore, dose comparisons between systems and modalities were summarized descriptively using proportions.

Generalized linear models (GLMs) were used for statistical comparisons of image quality (Table 1), with radiographic and fluoroscopic data analyzed separately. In each model, the response variable was regressed on a set of predictors (main effects) and their potential mutual dependencies (interaction terms). The regression coefficients signified the magnitude of the corresponding effects and could be tested for their statistical significance. The GLM framework does not assume normal error distributions, allowing it to be applied flexibly to both continuous (e.g., resolution) and discrete (e.g., contrast‐detail score) response variables.

**TABLE 1:** Generalized Linear Models used for statistical analysis of image quality data. All models included an intercept term, which is not explicitly displayed here in order to emphasize the main effects. Object sizes 1–6 were excluded from the contrast‐detail model analyses because all three systems demonstrated perfect detection of those objects.

Data	Generalized linear model	Main effect (levels)
Resolution, Radiographic^a^	*Resolution ∼ System + Direction + Mode*	System (OXOS/MinXray) Direction (AC/Trans) Mode (Handheld/Mounted)^b^
Resolution, Fluoroscopic	*Resolution ∼ System + Direction*	System (OXOS/OEC) Direction (AC/Trans)
Contrast‐Detail, Radiographic^a^	*Score_i_ ∼ System + Mode (i = 7, 8, 9, 10)*	System (OXOS/MinXray) Mode (Handheld/Mounted)^b^
Contrast‐Detail, Fluoroscopic	*Score_i_ ∼ System (i = 7, 8, 9, 10)*	System (OXOS/OEC)

Abbreviations: AC, the Anode‐Cathode direction of the X‐ray tube; Trans, the transverse direction of the X‐ray tube (perpendicular to the AC direction).

^a^ The OXOS MC2 DDR data were aggregated with radiographic data for model analysis.

^b^ The OXOS MC2 DDR data were acquired handheld.

To construct the most parsimonious model, we first evaluated the necessity of including the interaction terms by conducting a preliminary visual assessment. Marginal effect curves (average outcomes grouped by one predictor against another) were plotted to identify strong interactions, which manifest as “crossover” patterns between curves. As this visual assessment did not reveal such patterns, we concluded that all interaction terms were negligible. Therefore, only main effects were included in the final models to improve interpretability and statistical power. For the contrast‐detail curves, only the scores of the four smallest object sizes were compared, as all systems demonstrated perfect detection of larger objects.

The ten models listed in Table 1 included a total of 27 coefficients (including intercepts). To maintain an overall significance level (α) of 0.05, the significance threshold for each individual test was adjusted to 0.0019 (= 0.05/27) using the Bonferroni correction.^18^ Similar statistical approaches were employed for the inter‐modality and inter‐system comparisons of HVL, with further methodological details provided in the Supporting Information.

## 3 RESULTS

### 3.1 Primary, scatter, and leakage radiation measurement

**FIGURE 5:**
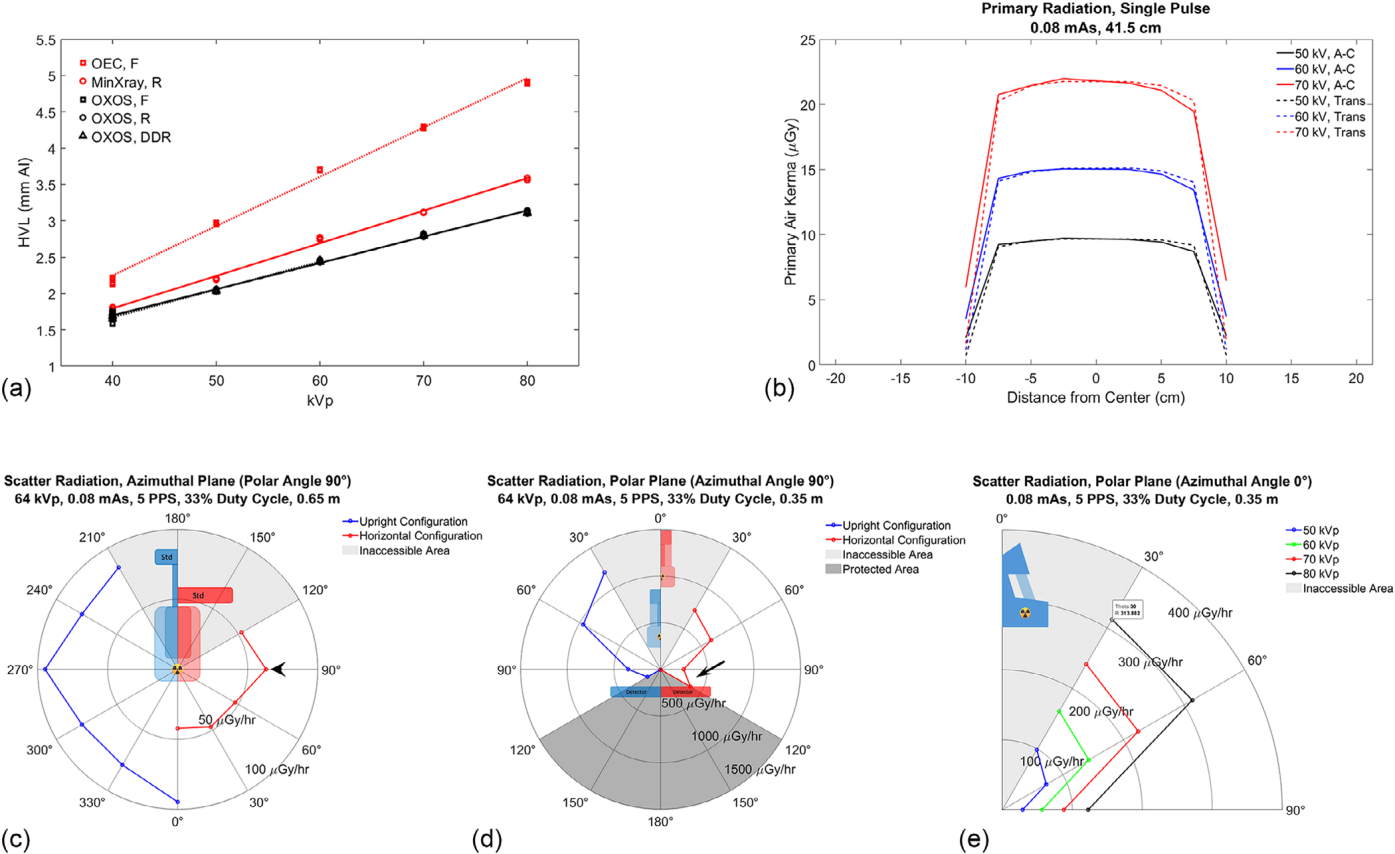
(a) Half‐value layer (HVL) of the system under study (OXOS) and two reference systems (MinXray and OEC). (b)‐(e) Primary and scatter radiation profiles of the OXOS MC2 system. The illustrations of system setup (X‐ray emitter, detector, and stand [std]) are provided for geometrical reference only and are not drawn to scale. (b) Primary radiation intensity profiles along the anode‐cathode direction (A‐C) and the perpendicular transverse direction (Trans) at three tube voltages. (c) Representative scatter profiles in the azimuthal (phantom) plane for the upright (left, blue) and horizontal (right, red) configurations. The scatter air kerma rate (AKR) is plotted in polar coordinates, where the radial distance from the origin represents the scatter AKR magnitude. (d) Representative scatter profiles in the polar (sagittal) plane, for the upright (left, blue) and horizontal (right, red) configurations. (e) Polar plane scatter profiles acquired at four different tube voltages.

The three systems demonstrated significantly different beam qualities across the evaluated kVp range, with the fluoroscopic reference exhibiting the highest HVL and the MC2 exhibiting the lowest (all *p* < 0.0001). Furthermore, the MC2 demonstrated consistent beam quality across all three of its imaging modalities (all *p* > 0.5; Figure 5a).

Representative examples of measured primary and scatter radiation profiles for the MC2 system are presented in Figure 5b‐e. Primary X‐ray intensity profiles across the Field of View (FOV) demonstrated spatial variations attributable to the heel effect and collimator cut‐off (Figure 5b). Although neither effect had a substantial impact on the visual appearance of the phantom images, both required appropriate correction during the semi‐automated contrast‐detail analysis (Figure 4).

Figures 5c,d display representative measured scatter profiles for the upright (left) and horizontal (right) configurations of the MC2 system in the azimuthal and polar planes, respectively. The horizontal configuration (SID: 55 cm) produced less scatter radiation than the upright configuration (SID: 45 cm). In the azimuthal plane, the distribution of scatter radiation was roughly isotropic. In the polar plane, intense scatter was observed only below a polar angle of 120°. Regions beyond this angle were shielded by the lead‐backed wireless detector. Increasing the tube voltage enhanced the overall scatter intensity while reducing the relative contribution of backscatter at low polar angles (Figure 5e).

Maximum tube leakage was measured on the lateral side of the X‐ray emitter. At maximum output (80 kVp, 0.4 milliampere‐seconds, [mAs]), a single pulse generated 6.1 nGy of leakage radiation at a distance of 10 cm from the focal spot. At the highest pulse rate (5 PPS) and duty cycle (33%), this corresponds to a leakage rate of 0.37 µGy per hour at one meter from the focal spot, well below the regulatory limit of 0.88 mGy per hour.^19^

### 3.2 Patient and operator dose estimate

Tables 2 and 3 summarize patient and operator dose estimates for the four extremity procedures evaluated. Procedural DAPs were generally consistent with values reported in the literature and established benchmarks.^20^, ^21^, ^22^, ^23^ The radiographic EAK of the MC2 system was substantially lower than that of the reference system (14%–59% of the reference). The fluoroscopic dose comparison demonstrated a higher level of heterogeneity. Depending on the exposure technique and patient positioning, the MC2's EAKR ranged from 15% to 245% of the fluoroscopic reference system's values across the four anatomical regions. The dose rates of the DDR were comparable to those of fluoroscopy (85%–124% of the MC2 fluoroscopic dose rate).

**TABLE 2:** Patient and operator dose comparison between the system under study (OXOS) and the radiographic reference system (MinXray).

Procedure		Hand (AP)		Shoulder (AP)		Ankle (AP/LAT)		Knee (AP/LAT)	
System		*MinXray*	*OXOS*	*MinXray*	*OXOS*	*MinXray*	*OXOS*	*MinXray*	*OXOS*
Patient Dose	EAK (mGy) ^a^	0.037	0.022	0.15	0.021	0.060	0.028	0.14	0.047
	DAP (mGy·cm^2^)	15	6	79	5	37	7	82	10
	DAP Reference (mGy·cm^2^)	112 (Meiboom^21^); 22 (UK NDRL^23^);		1464 (Meiboom^21^); 90 (UK NDRL^23^)		163 (Meiboom^21^)		1304 (Meiboom^21^); 54 (UK NDRL^23^)	
Operator Dose	Hp(10) (µSv)	0.213	0.068	0.619	0.054	0.104	0.047	0.223	0.074
	H_E_ (µSv)	0.038	0.012	0.110	0.010	0.019	0.008	0.040	0.013
	Hp(3) (µSv)	0.223	0.074	0.597	0.052	0.118	0.050	0.257	0.077
	Hp(0.07) (µSv)	0.219	0.073	0.569	0.049	0.121	0.049	0.267	0.076
	Leakage Contribution (%)	21.6%	0.3%	51.4%	0.7%	66.0%	0.3%	50.0%	0.2%
IRID (µGy) ^b^		9.15	2.10	33.91	3.25	15.00	4.41	36.24	7.02

Abbreviations: DAP, dose‐area product; EAK, entrance air kerma; HE, effective dose equivalent; IRID, image receptor input dose.

^a^ The following tissue thicknesses were assumed for EAK calculation: hand: 4 cm; shoulder: 14 cm; ankle: 8 cm; knee: 11 cm.

^b^ Measured using the ANSI extremity phantom and without patient table.

**TABLE 3:** Patient and operator dose comparison between the system under study (OXOS) and the fluoroscopic reference system (OEC).

Procedure		Hand (AP)		Shoulder (AP)		Ankle (AP/LAT)		Knee (AP/LAT)	
System		*OEC, F*	*OXOS, F (DDR)*	*OEC, F*	*OXOS, F* ^e^ *(DDR)*	*OEC, F*	*OXOS, F (DDR)*	*OEC, F*	*OXOS, F (DDR)*
Patient Dose	EAKR (mGy/min) ^a^	0.53	1.21 (1.21)	5.17	0.80 (1.00)	0.67	1.64 (1.40)	1.37	1.89 (2.35)
	DAP Rate (mGy·cm^2^/min)	469	345 (345)	3673	199 (247)	549	420 (359)	1046	420 (523)
	DAP (mGy·cm^2^) [Median Fluoro Time ^b^]	117 [0.25 min]	86 [0.25 min]	4151 [1.13 min]	224 [1.13 min]	137 [0.25 min]	105 [0.25 min]	122 [0.12 min]	49 [0.12 min]
	DAP Reference (mGy·cm^2^)	125 (Greffier DRL^22^)		1500 (Greffier DRL^22^)		205 (Greffier DRL^22^)		198 (Metaxas, median^20^)	
Operator Dose	Hp(10) Rate (µSv/hr)	441.5	351.4 (351.4)	5208.6	125.1 (160.3)	555.3	171.0 (140.5)	1257.6	171.0 (222.2)
	H_E_ Rate (µSv/hr) ^c^	78.8	62.7 (62.7)	930.1	22.3 (28.6)	99.2	30.5 (25.1)	224.6	30.5 (39.7)
	Hp(3) Rate (µSv/hr)	428.6	336.1 (336.1)	5079.2	119.0 (151.9)	558.1	180.7 (150.1)	1238.1	180.7 (231.9)
	Hp(0.07) Rate (µSv/hr)	407.7	318.4 (318.4)	4890.5	112.7 (144.1)	535.3	178.1 (148.4)	1184.1	178.1 (228.5)
	Leakage Contribution (%)	10.3%	0.1% (0.1%)	0.9%	0.6% (0.5%)	2.6%	0.3% (0.3%)	1.6%	0.3% (0.2%)
IDRIR (mGy/min) ^d^		0.108	0.184 (0.185)	1.171	0.128 (0.169)	0.130	0.274 (0.226)	0.296	0.274 (0.362)

Abbreviations: DAP, dose‐area product; DDR, Dynamic Digital Radiography; EAKR, entrance air kerma rate; F, fluoroscopy; HE, effective dose equivalent; IDRIR, input dose rate to image receptor.

^a^ The following tissue thicknesses were assumed for EAKR calculation: hand: 4 cm; shoulder: 14 cm; ankle: 8 cm; knee: 11 cm.

^b^ Median fluoro times from Greffier et al. for the hand, shoulder, and ankle procedures. Median fluoro time from Metaxas et al. for the knee procedure.

^c^ H_E_ was calculated from Hp(10) with conversion factor provided in NCRP Report No. 122.

^d^ Measured using the ANSI extremity phantom and without patient table.

^e^ Handheld fluoroscopy is not currently approved for clinical use in the United States. Therefore, the estimated shoulder fluoroscopic dose for the system under study (OXOS) represents a hypothetical scenario evaluated solely for academic and investigational purposes.

Compared to scatter radiation, tube leakage constituted a negligible component of operator dose for both the MC2 system and the fluoroscopic reference system, accounting for only 0.1%–10% of the total dose. Its contribution, however, was substantially greater with the radiographic reference system (21%–66% of the total dose). Both the radiographic and fluoroscopic metrics of occupational dose to the operator were markedly lower with the MC2 system. Specifically, the H_E_ per procedure was 9%–45% of the radiographic reference, and the H_E_ per unit time was 2%–80% of the fluoroscopic reference. The operator dose for DDR was comparable to that of fluoroscopy (82%–130% of the MC2 fluoroscopic operator dose).

The measured IRID and IDRIR exhibited a trend similar to that of the EAK and EAKR, with the MC2's radiographic IRID and fluoroscopic IDRIR ranging from 10% to 29% and from 11% to 211% of the corresponding reference system values, respectively.

### 3.3 Image quality assessment

**FIGURE 6:**
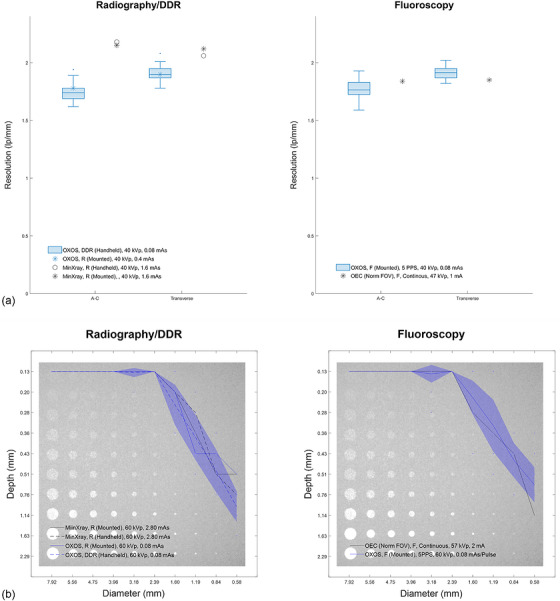
High‐contrast spatial resolution (a) and low‐contrast detectability (b) comparisons between the system under study (OXOS) and the reference systems, with the radiographic reference system (MinXray) on the left and the fluoroscopic system (OEC) on the right. The boxes and whiskers in (a) represent resolution distributions measured over multiple DDR or fluoroscopic frames along both anode‐cathode (A‐C) and transverse directions. The shaded areas in (b) represent mean ± one standard deviation of contrast‐detail scores obtained from multiple DDR or fluoroscopic frames.

High‐contrast spatial resolution of the three imaging systems is presented in Figure 6a. The MC2 system demonstrated identical spatial resolution in all three imaging modalities (1.8 lp/mm along the Anode‐Cathode direction, 1.9 lp/mm along the transverse direction), comparable to that of the fluoroscopic reference system (A‐C / trans: 1.8 / 1.9 lp/mm, *p* = 0.92) but significantly lower than that of the radiographic reference system (A‐C / trans: 2.1 / 2.2 lp/mm, *p* < 0.001).

The MC2 system also exhibited consistent low‐contrast performance across all three imaging modalities, as demonstrated by the contrast‐detail curves illustrated in Figure 6b. Its low‐contrast detectability was comparable to those of the two reference systems (*p* = 0.62, 0.23, 0.61, and 0.55 for the four smallest object sizes compared with the radiographic reference system; and *p* = 0.42, 0.79, 0.72, and 0.11 compared with the fluoroscopic reference system). Although a slight improvement in detectability of the finest details was observed with the MC2 system relative to the fluoroscopic reference system, the difference was not statistically significant (*p* = 0.11).

Both the MC2 system and the radiographic reference system can be operated either mounted on a cart or tripod, or handheld by the operator. In prolonged handheld operations, image quality might be negatively affected by operator fatigue, SID fluctuation, and tube instability. To assess the potential impact of these factors, we evaluated the influence of handheld operation on image quality. No measurable difference was observed in spatial resolution (*p* = 0.78). The contrast‐detail curves showed a slight reduction in low‐contrast detectability for the smallest objects during handheld operation. However, none of the results reached statistical significance after Bonferroni correction for multiple comparisons (*p* = 0.62, 0.36, 0.53, and 0.04 for the four smallest object sizes, respectively).

## 4 DISCUSSION

Compared with conventional imaging equipment, the MC2 system offers substantial improvements in versatility and portability for field deployment in remote and austere environments. Its X‐ray emitter is 51% lighter and 55% smaller by volume than the reference radiographic system. The complete system (including the wireless detector) weighs less than 5% of a full‐size C‐arm fluoroscopic system. This compact design, combined with battery‐powered operation, makes it possible to conduct fluoroscopic‐guided extremity procedures in regions without stable power grids or transportation infrastructure. Currently, external fixation without fluoroscopy is the standard of care in these settings due to the absence of fluoroscopic imaging equipment. The availability of lightweight, portable fluoroscopy could change the paradigm of field medicine by enabling additional treatment options and improving surgical outcomes.^11^

These improvements are primarily attributable to the MC2's adoption of a high‐sensitivity, real‐time image detector and a shorter SID. This configuration enables low‐mAs operation, which reduces energy consumption and enhances system reliability. However, these design innovations also raise concerns regarding potential compromises in image quality or radiation dose.

In the management of musculoskeletal injuries frequently encountered in field medicine, adequate soft‐tissue contrast is crucial for detecting internal infection or inflammation. Our data demonstrated that the MC2's fixed techniques generated comparable soft‐tissue contrast to that of the two reference systems, despite utilizing a lower input dose to the detector. Specifically, the MC2's IRID (0.81 µGy) was 13% of the reference radiographic system's value (6.23 µGy), while its fluoroscopic IDRIR (0.084 mGy/min) was 66% of the reference fluoroscopic system's AERC‐driven technique (0.128 mGy/min). These findings not only indicate sufficient low‐contrast performance for the MC2 protocols depicted in Figure 2 (with the exception of the hypothetical handheld shoulder protocol), but also suggest potential dose reduction opportunities for the MC2 hand and ankle fluoroscopic protocols, which utilize higher IDRIRs than those of the reference system (Table 3).

High‐contrast spatial resolution is most relevant for fracture detection. Our data demonstrated that the MC2 system provides fluoroscopic spatial resolution comparable to a full‐size C‐arm, despite having a larger nominal focal spot (0.8 vs. 0.3). However, the radiographic resolution of both the MC2 and the portable reference were below the established criteria for optimal fracture detection with stationary digital radiography.^24^, ^25^ This finding was consistent with the general image quality trade‐offs inherent to portable radiography.^26^, ^27^ The spatial resolution of the MC2 system was slightly lower than that of the reference system. Since both systems have identical nominal focal spot and detector pixel pitch, this difference was attributed to increased geometric blurring resulting from the MC2's shorter SID.

However, the clinical impact of this reduction may be limited. Murphey et al. reported that when the spatial resolution of digitized screen‐film radiography decreased from 2.88 lp/mm to 1.44 lp/mm, the area under the Receiver Operating Characteristic (ROC) curve for detecting nondisplaced extremity fractures reduced only from 0.83 ± 0.03 to 0.81 ± 0.03.^25^ Therefore, while the reduced radiographic resolution of the MC2 system might have a small negative impact on the detection of subtle fractures, its influence is likely to be negligible in the management of larger, complex factures.

The finding that the MC2 system has relatively low HVL is consistent with its light construction. Both its softer beam and shorter SID result in a higher patient skin dose, particularly in fluoroscopic procedures. Nevertheless, its fluoroscopic dose rates across all procedures remained well below the regulatory limit (44 mGy/min for fluoroscopic systems without AERC), even at the minimum SSD (3.53 mGy/min). The risk of radiation‐induced skin burns is negligible, especially when the surgical risks of operating without fluoroscopic guidance in austere environments are considered.

Conversely, the radiographic reference system, despite having a longer SID and higher HVL than the MC2, still generated higher EAKs for all evaluated procedures. This suggests that its recommended technical parameters may not be fully optimized for high‐sensitivity digital detectors, whereas technical and post‐processing optimizations targeted to the a‐Si detector enabled the MC2 system to achieve more pronounced mAs reduction.

Regarding operator safety, the compact dimensions of the MC2 system enable flexible patient setup, thus facilitating occupational dose reduction by allowing the operator to stay in regions with relatively low scatter radiation. Our data showed that all operator dose metrics estimated for the four anatomical regions were consistently lower than those of the reference systems for both radiographic and fluoroscopic imaging. Moreover, features such as the exposure interlock function, typically absent in existing portable radiographic systems, further enhance radiation safety by reducing the risk of accidental exposure in poorly controlled field environments.

While DDR is currently classified in the United States as a form of general‐purpose radiography, its rapid, repeated exposure pattern resembles a pulsed fluoroscopic series. Our data demonstrated that the dose rates associated with DDR are comparable to those of fluoroscopy, suggesting that similar safety measures, including personnel training, dose monitoring, and radiation protection measures, should be applicable to both imaging modalities.

Although this study focused on field imaging applications in remote and austere environments, DDR imaging equipment also requires special shielding considerations if used in hospital‐based settings. For this purpose, the MC2 system can be treated similarly to a conventional fluoroscopy system, as the presence of the interlock function prevents direct exposure of structural barriers to the primary beam. Existing examination rooms designed for C‐arm procedures should provide adequate shielding, provided that the combined workload of all three modalities does not exceed the room's design limit. However, this assessment should not be generalized to all DDR systems. Systems designed for pulmonary applications, for example, exhibit higher tube output and larger FOV.^28^ In general, shielding for examination rooms hosting DDR equipment should be determined on a case‐by‐case basis.

The MC2 system imposed a 20‐second limit on the maximum duration of fluoroscopic and DDR series. In practice, this limit is unlikely to pose a serious constraint for the system's intended use, as orthopedic procedures seldom utilize extended exposures. In a recently published five‐year retrospective study, Metaxas et al. reported fluoroscopy time ranges of 2.00–38.0 s for hand, 1.60–23.0 s for ankle/foot, and 2.85–45.0 s for knee procedures.^20^ Since these are total fluoroscopy times composed of multiple shorter exposures, the 20‐second single‐exposure limit would be sufficient for the majority of cases.

Presently, there is no consensus on the optimal temporal resolution for fluoroscopy‐guided orthopedic procedures, and clinical practices vary substantially across institutions. The MC2 system features a maximum pulse rate of 5 PPS. While this temporal resolution is on the lower end of common fluoroscopic frame rate (2‐15 PPS), Genetay et al. demonstrated its practicality for the majority of common orthopedic trauma cases.^29^ The frame rate is also likely to be adequate for emerging DDR orthopedic applications. Unlike pulmonary imaging, which requires higher pulse rates (up to 15 PPS) to capture rapid, involuntary respiratory motion,^6^ orthopedic DDR typically involves slower, voluntary movements of the joints that can be resolved at lower temporal resolutions.

Our study has several limitations. First, all image quality evaluations were phantom‐based. While standardized phantom tests are reproducible, the clinical relevance of the testing results is not always well established. Further prospective clinical studies are needed to establish the precise clinical impact of our findings, particularly regarding reduced radiographic spatial resolution. Future work should also investigate the implications of this blurring for automated image processing in orthopedic and multi‐center settings.

Second, the patient setups used for dose evaluation were not exhaustive. However, they provide conservative estimations that can serve as readily available references for quick estimation in the field. Had a more refined dose estimate been needed, the methods employed in this study are sufficiently versatile to be adapted to any new patient setups encountered in clinical practice. Despite the limitation in setup variety, our data provided sufficient evidence of the MC2's safety. The highest fluoroscopic H_E_ rate was recorded with the hand procedure at 62.7 µSv/hr. At this dose rate, a user could theoretically operate the MC2 system *continuously* for nearly 800 h before reaching the U.S. Nuclear Regulatory Commission's annual occupational dose limit of 50 mSv. Given the intermittent nature of X‐ray use in extremity procedures, such prolonged exposure is highly unlikely in clinical practice.

Third, while the average clinical techniques used in the analysis above were representative of common practice in orthopedic clinics, they may not reflect the most dose‐efficient protocols for the reference fluoroscopy system. For instance, an orthopedic surgeon could have achieved lower operator dose with pulsed rather than continuous fluoroscopy. The dose‐saving potential of pulsed fluoroscopy is limited in extremity procedures, though. Due to the short exposures typically employed in these procedures, the large AEC pre‐pulse (700 ms) often overwhelms the few imaging pulses and dominates the total radiation output. Our measurements showed that pulsed fluoroscopy at 8 PPS reduced scatter radiation by approximately 40% for 1‐second exposures and 60% for 5‐second exposures. Even with this level of dose reduction, the operator dose of the reference C‐arm remained unfavorable compared to the MC2 system for 75% of procedures.

Finally, the “virtual badge” method used for operator dose estimation has limitations. While adaptable, its reliance on multiple published conversion factors increases uncertainty through error propagation, leading to lower precision compared with alternative methods based on Monte Carlo simulation or direct measurement using anthropomorphic phantoms. Furthermore, spatial discrepancies between the virtual badge and radiosensitive organs can compromise accuracy. For example, Tanaka et al. reported Hp(3)/Hp(10) ratios obtained from direct measurement at the surgeon's eye level were lower than those derived from the ENEA conversion factors and were height‐dependent.^30^ These findings suggest that Hp(3) estimates obtained with the “virtual badge” method may be overestimated. These limitations should be taken into consideration when applying dose estimates derived from this method.

In conclusion, we conducted a systematic comparison of radiation dose and image quality between a portable, multi‐modality extremity imaging system and conventional radiographic and fluoroscopic equipment designed for similar intended uses. Despite compromises in radiographic resolution and fluoroscopic EAKR, the OXOS MC2 system maintained image quality largely comparable to the benchmark systems, while operating well below applicable regulatory dose limits. The radiation doses associated with the DDR modality were found to be comparable to or lower than those of conventional fluoroscopy, indicating no significant safety concerns. Overall, this system provides a safe and versatile imaging solution for the resource‐limited environments frequently encountered in field and emergency medicine. By enabling state‐of‐the‐art fluoroscopy and image‐guided orthopedic procedures in previously inaccessible regions, the MC2 system holds the potential to significantly improve trauma patient triage and surgical management in remote and austere environments.

## CONFLICT OF INTEREST STATEMENT

This study was sponsored by OXOS Medical, Inc., the manufacturer of the device under study. Acknowledged contributors Tom Lombardo and Jonathan Shaw were employees of OXOS Medical, Inc. at the time of the study.

## Supporting information



Supporting Information

## References

[1] Clement David‐Olawade A, Olawade DB , Vanderbloemen L , et al. AI‐driven advances in low‐dose imaging and enhancement—a review. Diagnostics. 2025;15(6):689. doi:10.3390/diagnostics15060689 40150031 PMC11941271

[2] Alharbi MKM , Zhou A , Naunton M , Davidson R , Makanjee C . Innovations in X‐ray tube design and instrumentation for conventional radiological applications: a scoping review. Imaging Sci J. 2025;73(6):653‐668. doi:10.1080/13682199.2025.2470485

[3] Beer Y , Shabshin N , Copel L , et al. Evaluation of the diagnostic potential of a Tomosynthesis system for MSK. ECR 2025 Book of Abstracts. Springer Science and Business Media LLC; 2025:294. doi:10.1186/s13244-025-02003-8

[4] Melhem E , Assi A , El Rachkidi R , Ghanem I . EOS biplanar X‐ray imaging: concept, developments, benefits, and limitations. J Child Orthop. 2016;10(1):1‐14. doi:10.1007/s11832-016-0713-0 26883033 PMC4763151

[5] Calabrò E , Lisnic T , Cè M , Macrì L , Rabaiotti FL , Cellina M . Dynamic digital radiography (DDR) in the diagnosis of a diaphragm dysfunction. Diagn Basel. 2024;15(1):2. doi:10.3390/diagnostics15010002 PMC11720026 39795531

[6] Fyles F , FitzMaurice TS , Robinson RE , Bedi R , Burhan H , Walshaw MJ . Dynamic chest radiography: a state‐of‐the‐art review. Insights Imaging. 2023;14(1):107. doi:10.1186/s13244-023-01451-4 37332064 PMC10277270

[7] Upasani VV , Bandaralage H , Farnsworth CL . 3D cone‐beam tomosynthesis provides axial imaging of the spine with lower radiation compared to computed tomography. Spine Deform. 2021;9(1):41‐49. doi:10.1007/s43390-020-00199-x 32930997

[8] Zouris JM , Wade AL , Magno CP . Injury and Illness casualty distributions among U.S. army and marine corps personnel during operation Iraqi freedom. Mil Med. 2008;173(3):247‐252. 18419026 10.7205/milmed.173.3.247

[9] Belmont PJ , Owens BD , Schoenfeld AJ . Musculoskeletal injuries in Iraq and Afghanistan: epidemiology and outcomes following a decade of war. J Am Acad Orthop Surg. 2016;24(6):341‐348. doi:10.5435/JAAOS-D-15-00123 27115793

[10] Wojcik BE , Humphrey RJ , Czejdo B , Harrison Hassell L . U.S. army disease and nonbattle injury model, refined in Afghanistan and Iraq. Mil Med. 2008;173(9):825‐835. 18816921 10.7205/milmed.173.9.825

[11] Bar‐On E , Herard P . Orthopedics in a field hospital. In: Bar‐On E , Peleg K , Kreiss Y , eds. Field Hospitals. 1st ed.. Cambridge University Press; 2020:180‐195. doi:10.1017/9781316493489.018

[12] AAPM . Standardized Methods for Measuring Diagnostic X‐Ray Exposures (AAPM Report No. 31). American Association of Physicists in Medicine; 1990.

[13] Gualdrini GF , Morelli B . Air kerma to personal dose equivalent conversion factors for the ICRU and ISO recommended slab phantoms for photons from 20 keV to 1 MeV. ENEA; 1996.

[14] NCRP . Use of Personal Monitors to Estimate Effective Dose Equivalent and Effective Dose to Workers for External Exposure to Low‐LET Radiation: Recommendations of the National Council on Radiation Protection and Measurements (NCRP Report No. 122). National Council on Radiation Protection and Measurements; 1995.

[15] Lin PP , Goode AR , Corwin FD , et al. AAPM task group report 272: comprehensive acceptance testing and evaluation of fluoroscopy imaging systems. Med Phys. 2022;49(4):e1‐e49. doi:10.1002/mp.15429 35032394

[16] Samei E , Flynn MJ , Reimann DA . A method for measuring the presampled MTF of digital radiographic systems using an edge test device. Med Phys. 1998;25(1):102‐113. doi:10.1118/1.598165 9472832

[17] Scott AW , Zhou Y , Zhang D , Binesh N , Lee C , Bosteder M . Dose reduction in digital radiography based on the significance of marginal contrast detectability. J Appl Clin Med Phys. 2021;22(5):117‐127. doi:10.1002/acm2.13230 PMC8130234 33773008

[18] Dunn OJ . Multiple comparisons among means. J Am Stat Assoc. 1961;56(293):52‐64. doi:10.1080/01621459.1961.10482090

[19] 21 C.F.R § 1020.30 . U.S. Food and Drug Administration; 2025.

[20] Metaxas VI , Savvakis S , Skouridi E , et al. Typical dose values for intra‐operative fluoroscopy during orthopaedic trauma surgery at Larnaca general hospital in cyprus: a five‐year retrospective study. Injury. 2025;56(2):112089. doi:10.1016/j.injury.2024.112089 39724809

[21] Meiboom MF , Hoffmann W , Weitmann K . Tables for effective dose assessment from diagnostic radiology (period 1946–1995) in epidemiologic studies. PLoS One. 2021;16:e0248987. doi:10.1371/journal.pone.0248987 33793615 PMC8016243

[22] Greffier J , Etard C , Mares O , et al. Patient dose reference levels in surgery: a multicenter study. Eur Radiol. 2019;29(2):674‐681. doi:10.1007/s00330-018-5600-2 30069810

[23] National Diagnostic Reference Levels (NDRLs) from 8 July 2025 . UK Health Security Agency. 2025. Accessed October 20, 2025. https://www.gov.uk/government/publications/diagnostic‐radiology‐national‐diagnostic‐reference‐levels‐ndrls/ndrl

[24] Jonsson Á , Laurin S , Karner G. et al., Spatial resolution requirements in digital radiography of scaphoid fractures: An ROC analysis. Acta Radiol. 1996;37(4):555‐560. doi: 10.1177/02841851960373P226 8688242

[25] Murphey MD , Bramble JM , Cook LT , Martin NL , Dwyer SJ . Nondisplaced fractures: spatial resolution requirements for detection with digital skeletal imaging. Radiology. 1990;174(3):865‐870. doi:10.1148/radiology.174.3.2305071 2305071 10.1148/radiology.174.3.2305071

[26] Zakaria I , Yus TM , Rahman S , Gani A , Ersan MA . Assessing fracture detection: a comparison of minimal‐resource and standard‐resource plain radiographic interpretations. Diagnostics. 2025;15(7):876. doi:10.3390/diagnostics15070876 40218227 10.3390/diagnostics15070876 PMC11988379

[27] Strickland LR , Henry TS , McAdams HP , Tailor TD , O'sullivan‐Murphy B , Heyneman LE . Portable chest radiography: must‐know findings and mimics. Radiographics. 2023;43(9):e220132. doi:10.1148/rg.220132 37651275 10.1148/rg.220132

[28] Siddique A , Ge G , Zhang J . Radiation dose and shielding considerations for digital dynamic radiography (DDR) compared to mobile C‐arms. J Appl Clin Med Phys. 2025;26(9):e70256. doi:10.1002/acm2.70256 40903809 10.1002/acm2.70256 PMC12408374

[29] Genetay T , Gamulin A , Lorimier A , Sans Merce M . Assessment of radiation dose values in common orthopaedic trauma examinations performed under X‐ray fluoroscopy guidance. Radiat Prot Dosimetry. 2024;200(14):1365‐1371. doi:10.1093/rpd/ncae175 39193892 10.1093/rpd/ncae175 PMC11384882

[30] Tanaka T , Matsubara K , Fukuda A , Kobayashi S . Estimation OF Hp(3) to the eye lens of interventional radiologists‐relation between the eye lens dose and radiologist's height. Radiat Prot Dosimetry. 2019;187(4):409‐417. doi:10.1093/rpd/ncz181 31605136 10.1093/rpd/ncz181

