# Supplementary material for: A Comparative dose and image quality assessment of a portable, multi‐modality extremity imaging system for austere environments
Source: Med Phys. 2026 Jul 25;53(8):e70574. doi: 10.1002/mp.70574 (PMC13401090; doi:10.1002/mp.70574)
Supplement: Supplementary file 1 — Supporting Information [file MP-53-0-s001.pdf]

Supplementary Materials

S1. Measurement Methodology for Image Receptor Input Dose (IRID) and Input Dose Rate (IDRIR)

Transmitted radiation dose (D, for radiography) or dose rate ($\dot{D}$, for fluoroscopy and DDR) was measured behind a phantom in the clinical configurations depicted in **Figure 2**, with the dosimeter on the surface of the image receptor and the phantom placed flush to the dosimeter. The measured dose or dose rate was then corrected for distance and grid transmission:

$$IRID = D \times \left(\frac{SDD}{SID}\right)^2 \times T$$

$$IDRIR = \dot{D} \times \left(\frac{SDD}{SID}\right)^2 \times T$$

where SID is the source-to-image distance specified in **Figure 2**, and SDD is the distance from the source to the dosimeter. Specifically, SDD was 99 cm for all OEC and MinXray configurations, and 43, 78, 54, and 54 cm for the OXOS hand, shoulder, knee, and ankle configurations, respectively. A transmission factor (T) of 0.72 was used for the OEC C-arm system according to the manufacturer's specification. Since neither the OXOS nor the MinXray system is equipped with a built-in grid, a T value of 1 was adopted for all of their configurations.

The IRID and IDRIR values reported in **Tables 2 and 3** were measured with the ANSI Extremity phantom and did not account for additional patient support devices. We also measured the combined effects of patient table attenuation and scatter using an OEC C-arm. For tube voltages ranging from 54 to 74 kVp, introducing a typical fluoroscopic procedural table into the beam led to a 24-28% decrease in IDRIR.

The IRID and IDRIR values reported in the Discussions section in the context of low-contrast performance were measured using the Gammex contrast-detail phantom utilized in our low-contrast assessment.

S2. Statistical Approach for Inter-Modality and Inter-System Comparisons of HVL

HVL values were measured at tube voltage levels of 40, 50, 60, 70, and 80 kVp for the OEC fluoroscopic, MinXray radiographic, and OXOS radiographic and DDR modalities. For the OXOS fluoroscopic modality, data were only acquired at 40, 50, and 60 kVp because the maximum

fluoroscopic tube voltage was limited to 64 kVp. Four measurements were obtained for each kVp station.

Due to the unbalanced data structure resulting from the limited OXOS fluoroscopic kVp range, a GLM approach was preferred over an Analysis of Variance (ANOVA). The following GLM was first applied to the OXOS data to assess inter-modality differences in HVL:

$$HVL_{OXOS} \sim kVp + Modality$$

where kVp is a continuous independent variable, and Modality is a discrete independent variable with three levels: Radiography, Fluoroscopy, and DDR.

Since both graphical (**Figure 5a**) and GLM analyses failed to demonstrate a significant dependence of the MC2's HVL on imaging modality (p = 0.57 for DDR vs. Radiography and p = 0.67 for Fluoroscopy vs. Radiography), we pooled all three OXOS modalities for the inter-system comparison using the following GLM:

$$HVL \sim kVp + System$$

where System is a discrete independent variable with three levels: OXOS, OEC, and MinXray.

Both GLMs estimate four coefficients (including the intercept). Therefore, the significance threshold for each individual coefficient was adjusted to 0.00625 (0.05/8) using the Bonferroni correction.
